# When Work Conflicts With Personal Projects: The Association of Work-Life Conflict With Worker Wellbeing and the Mediating Role of Mindfulness

**DOI:** 10.3389/fpsyg.2021.539582

**Published:** 2021-11-08

**Authors:** Tyler Pacheco, Simon Coulombe, Sophie Meunier

**Affiliations:** ^1^Department of Psychology, Wilfrid Laurier University, Waterloo, ON, Canada; ^2^Département des Relations Industrielles, Université Laval, Quebec City, QC, Canada; ^3^Vitam – Centre de Recherche en Santé Durable, Quebec City, QC, Canada; ^4^Département de Psychologie, Université du Québec à Montréal, Montreal, QC, Canada

**Keywords:** work-life conflict, workplace, mental health, personal projects, mindfulness

## Abstract

The negative emotional and health effects of work-life conflict (WLC) have been demonstrated in numerous studies regarding organizational psychology and occupational health. However, little is known about WLC’s relationship with positive wellbeing outcomes, including emotional, psychological, and social aspects of workers’ thriving. Furthermore, the mediating processes underlying the effects of WLC remain mostly unknown. The current study investigated the associations of perceived time- and strain-based WLC with positive mental health and thriving at work, as well as the mediating role of mindfulness in these associations. It is argued that WLC causes reduced mindfulness capacities among workers, which is in turn associated with lower positive wellbeing given the importance of mindfulness in emotion regulation. A sample of 330 workers based in Québec, Canada, completed an online survey including a measure of strain- and time-based interference with personal projects (i.e., the goals and activities that define the daily life of an individual) and validated scales of wellbeing outcomes and mindfulness. Results of structural equation modeling revealed negative associations between time- and strain-based WLC with positive mental health and thriving at work. Work-life conflict was related to lower mindfulness, which played a mediating role in the associations between time-based WLC with positive mental health and thriving at work, as well as strain-based WLC with positive mental health. The mediation was complete for the time-based WLC and positive mental health association, but partial for the other mediated pathways, highlighting the need for more research to identify additional mediators. These results highlight that beyond resulting in negative emotional/health outcomes often studied in previous research, WLC may be associated with workers’ reduced potential to live a fulfilling life, in general and in the workplace. Recommendations (e.g., mindfulness intervention to promote emotional regulation, personal project intervention) for workplace policymakers and practitioners are identified.

## Introduction

In 2016, 47% of working Canadians considered their work to be the most stressful part of daily life (Canadian Centre for Occupational Health and Safety, 2016). Canadian workers also reported work-related stress as the primary cause of their mental health concerns (Shepell and Mental Health Commission of Canada, 2018). Approximately 500,000 Canadian workers take sick leave from work within a given week (Centre for Addiction and Mental Health, n.d.). In European countries, work-life conflict (WLC) has been shown to have maladaptive effects on both mental and physical health (Borgmann et al., 2019). Evidence from German and Austrian samples, for example, have supported the relationship between WLC and mental health concerns, such as depression (Haggag and Geser, 2012; du Prel and Peter, 2015). Of the stressors that can cause mental health concerns, researchers have found that forms of WLC (e.g., time- and strain-based conflicts) negatively affect workers’ emotional wellbeing and health (Haar et al., 2014). However, more research is needed to understand the psychological processes explaining how WLC influences wellbeing. By identifying these processes, interventions to counteract these processes can be developed and implemented in order to support workers’ wellbeing. This research explores the association between WLC and workers’ positive mental health (including positive emotions) and workplace wellbeing in Québec, Canada, as well as the mediating role of mindfulness in that relationship.

### Work-Personal Project Conflict and Workers’ Wellbeing

The relationship between work and personal life is generally viewed from two different perspectives, the first being the conflict that workers experience between various work and life demands (Barnett, 1998; Reynolds, 2005) and the second being their ability to balance these demands, thus achieving a higher degree of wellbeing (Reynolds, 2005). In the present article, a work-life conflict perspective is used given that the larger study in which this article is nested included a measure of that construct.

Work-life conflict is characterized by the conflicting roles required by the organization someone is employed by and their own family or personal life (Aslam et al., 2011). Work-life conflict includes two important dimensions, strain- and time-based conflict. As described by Greenhaus and Beutell (1985), time-based conflict refers to the multiple roles that compete for a worker’s time. In this dimension, time pressures that are associated with one’s role (e.g., worker) may make it difficult to comply with the demands in other roles (e.g., parent, partner). Previous research has illustrated the connection of work-related time commitment with WLC, as work-family conflicts increase with working more in a given week (Pleck et al., 1980; Greenhaus and Beutell, 1985). Strain-based WLC refers to the fact that stressors in one domain (e.g., work) lead to negative emotions and physical strain (e.g., tension, anxiety, depression, energy depletion), which reduces one’s capacity to accomplish their roles in the other domains (e.g., personal life; Greenhaus and Beutell, 1985; Steiber, 2009; Kirby, 2017).

When examining conflict between work and personal life, a significant amount of research has focused specifically on work-family conflicts and how demands in the workplace can clash with the demands of family roles (e.g., parent, spouse; Kossek and Lee, 2017). However, exploring work-family conflicts may not always be the most appropriate, as some workers may be single or not have children (Keeney et al., 2013). In comparison to work-family conflict, WLC explores the tensions between work demands and the array of other roles in workers’ lives, including family-related roles, but also roles in other areas such as friendship, self-care, education, and community participation (Kossek and Lee, 2017). Work-life conflict has been related to a greater likelihood of experiencing mental health concerns, even when considering the contributions of other established factors that are associated with impacting workers’ wellbeing (i.e., job characteristics such as demands, control, and support; Neto et al., 2018). Scales used to measure work-life conflict usually include relatively generic items exploring the impacts of work demands on home activities, relationships with friends and family, and a vast array of other life domains outside of work (Keeney et al., 2013). While the findings gained with generic measures are insightful, in the current study, we argue for an idiosyncratic approach that considers the unique goals and interests that matter for each individual, i.e., their personal projects. Varying from one person to another, personal projects are the goals and activities that define the daily life of an individual (Little, 2014, 2017; Little and Coulombe, 2015). The sustainable pursuit of one’s idiosyncratic personal projects has been shown to be associated with higher levels of sense of meaning and happiness (Little, 2014, 2017; Little and Coulombe, 2015). As such, interference of work with personal projects is likely to have an important toll on one’s mental health. Although past research has suggested that an approach for measuring work-life conflict with a focus on personal projects may be useful (Grawitch et al., 2011), it has rarely been implemented, to our knowledge, in empirical studies. A study conducted by Wiese and Salmela-Aro (2008) explored interference between work goals and personal goals. However, the latter were focused on family-related personal goals specifically. Focusing on work interference with what really matters to a person (whether it is family-related or related to other important pursuits, e.g., leisure, self-development, community involvement) has the potential to provide a more nuanced and ecologically valid representation of how WLC affects worker wellbeing.

### Wellbeing as More Than the Absence of Mental Health Concerns

Keyes and colleagues (Keyes, 2005; Provencher and Keyes, 2011) have discussed the complexity of mental health as a holistic construct. Their work suggests that complete mental health should be assessed on two dimensions that are only moderately correlated: the absence of mental health concerns (i.e., negative symptoms such as depression and anxiety), and the presence of positive mental health, including positive emotions (e.g., satisfaction, interest), psychological functioning (e.g., purpose, autonomy), and social functioning (e.g., social integration, social contribution). In a study by Coulombe et al. (2016), and contrary to expectations, some people living with common mental health concerns displayed relatively high levels of positive wellbeing despite dealing with significant symptoms of anxiety or depression at the same time. This exemplifies a certain level of independence between the two dimensions of mental health, and the inability to determine one’s level of positive wellbeing solely from measures evaluating negative wellbeing.

Based on these findings, it is evident that conclusions about the relationship between WLC and wellbeing should not be made just by exploring indicators related to negative aspects of wellbeing. Rather, a holistic perspective is crucial. Based on a recent review of the literature on WLC and its outcomes (Gisler et al., 2018), a diverse range of negative outcomes have been considered, such as burnout, anxiety, and depression, while the considered positive mental health indicators (i.e., positive emotions, job satisfaction) seem to be more limited.

### Mindfulness and Its Potential Mediating Role

Summarizing the findings of their review on work-life conflict research, Kossek and Lee (2017) identified that while there is evidence on the antecedents and consequences of WLC, there is still a scarcity of research exploring mediators in this body of research. In the current research, we focus on mindfulness as a potential mediator of the effect of WLC on wellbeing outcomes.

Mindfulness refers to “the awareness that emerges through paying attention on purpose, in the present moment, and non-judgmentally to the unfolding of experience moment by moment” (Kabat-Zinn, 2003, p. 145). As a personal disposition, it has been understood as a cognitive style that supports being aware of one’s thoughts and emotions and actively engaging in “being” instead of “doing” (Kostanski and Hassed, 2008). In the current study, we consider mindfulness specifically in the workplace, representing the degree to which one is mindful specifically in their work setting(s) (Dane and Brummel, 2014). As expressed by Dane and Brummel (2014), this degree is considered to be tied to one’s general mindfulness disposition in life, but it is also impacted by other factors, such as contextual elements of work that may “cue” mindfulness for some people.

While we focus our attention on mindfulness as a “state or quality of mind” (Lomas et al., 2017, p. 492), it is also important to note that the term mindfulness is also frequently used to refer to meditation practices or interventions intended to cultivate mindfulness skills, either in a formal therapeutic context or not (see also Glomb et al., 2011). Although the current study does not focus on a mindfulness intervention, findings from such studies are important to note given that they demonstrate that one’s level of mindfulness may change. For example, interventions have been associated not only with increased experiences of state mindfulness, but also with positive changes in participants’ mindfulness dispositions (Kiken et al., 2015; Tang, 2017). Interestingly, although mindfulness can be considered a trait (i.e., disposition) as in the current study, it has been highlighted to be more malleable than other individual differences (Xu et al., 2021). We will argue later that mindfulness levels at work could increase or decrease depending on one’s levels of WLC.

Recent reviews of workplace- and organizational-focused research suggests that mindfulness is associated with positive outcomes for workers and organizations (Lomas et al., 2017; Passmore, 2019). A key process underlying the positive association of mindfulness with positive outcomes is the emotion regulation enhancement associated with higher mindfulness (Guendelman et al., 2017). A few studies have focused on mindfulness in relationship with WLC (or work-life balance); however, these studies explored mindfulness primarily as an intervention rather than a natural state or quality. Mindfulness-based training interventions (i.e., cognitive, emotional, behavioral) have been shown to be associated with improvement of work-family balance (Michel et al., 2014; Kiburz et al., 2017). In a study by Michel et al. (2014), 208 participants received training on exercises related to mindfulness-based cognitive therapy and mindfulness-based stress reduction. Findings indicate that the training positively affected WLC by alleviating their struggles with workplace-related negative cognitions and emotions, as well as energy depletion (Michel et al., 2014). Further, the effects of mindfulness on work-family conflict have been demonstrated by Kiburz et al. (2017) as workers who received mindfulness-based workshops showed a decrease in work-family conflict. These two studies explored the effectiveness of a mindfulness intervention on WLC. However, for the current study, we expand on this work by focusing on mindfulness at work as a cognitive skill that different workers exhibit to varying degrees, in their natural work environment, without an intervention (Dane and Brummel, 2014).

In the workplace context, mindfulness involves workers being conscious of the internal and external stimuli related to the efforts they deploy in their work (Dane and Brummel, 2014; Herda et al., 2019). There is formative research evidence suggesting that mindfulness could play a role in the relationships between WLC and workers’ wellbeing outcomes. First, there is a substantial body of evidence supporting a positive association between levels of mindfulness and workers’ positive outcomes, including higher work performance, work satisfaction, quality of life, and resilience. There is also an association with reduced anger, anxiety, depression, stress, and burnout (see reviews by Good et al., 2016; Lomas et al., 2017; Mesmer-Magnus et al., 2017).

Secondly, increased WLC may be associated with reduced levels of mindfulness. However, to our knowledge, no research has been conducted with a direct focus on the relationship between WLC and workers’ mindfulness. As stated prior, in previous research, mindfulness has mostly been studied as an intervention to reduce WLC, rather than as a cognitive style that should be considered in non-intervention contexts. However, it has been argued that although it has its roots in dispositional mindfulness, workplace mindfulness is likely conditioned (reduced or amplified) by organizational factors (Dane and Brummel, 2014; Reb et al., 2015). Reb et al. (2015) argued that when constrained by negative organizational factors or stressors, workers will experience higher stress and will devote their mental energy to dealing with the stressors, thereby depleting their energy reserve, which is essential to enable being mindful at work. Similarly, Lawrie et al. (2018) explained that psychological demands related to energy loss, time pressure, and thoughts being absorbed by multiple deadlines at the same time would lead to the diminution of the cognitive resources necessary to monitor and focus their attention and awareness, which is key to experiencing mindfulness. The two groups of authors just mentioned found empirical evidence that organizational stressors (e.g., job demands such as requirements to work very hard; Lawrie et al., 2018; poor equipment, inadequate training, conflicting job demands, Reb et al., 2015) relate to lower levels of mindfulness (measured as a trait or a state depending on the study). In another study, busyness and fatigue (in general, not workplace-specific) was found to be significant negative predictors, respectively, of momentary awareness (i.e., being aware of present moment experience) and non-reactivity (i.e., having a non-elaborative, open, and accepting orientation), two aspects involved in mindfulness processes (Suelmann et al., 2018). In the workplace context of the current study, it is plausible to think that, over time, WLC as a workplace organizational factor having an impact on both busyness (time-based WLC) and fatigue (strain-based WLC) would impede the possibility to devote cognitive resources to attention focus, awareness, and non-reactivity, hereby reducing workplace mindfulness.

This is also aligned with several studies suggesting a negative association of stress with one’s level of mindfulness. For example, Crosswell et al. (2019) recently published findings suggesting that higher chronic stress levels were associated with greater levels of mind wandering, a construct negatively related with mindfulness (Mrazek et al., 2012). Another study found that workload was associated with lower levels of mindfulness (Hülsheger et al., 2018). The mediating role of mindfulness that is proposed in the current research is also aligned with a dissertation study conducted with teachers that identified mindfulness as playing a statistically significant (albeit small) mediating role in the relationship between perceived stress and burnout (Hanley, 2017). Finally, a study focusing on the construct of work-family conflict (Davis et al., 2017) showed that the effect of conflict on several outcomes (i.e., life satisfaction, positive and negative affect, perceived health) was mediated by the experiences of repetitive and intrusive thoughts. Although the study did not measure mindfulness *per se*, it is reasonable to extrapolate the study findings’ applicability to the notion of mindfulness as intrusive thoughts are associated with lower mindfulness scores (Catak, 2012) and reduced rumination is considered a key mechanism underlying mindfulness (Svendsen et al., 2017).

When considering the established connections between mindfulness and workers’ wellbeing, a negative relationship between WLC and mindfulness may translate into more negative wellbeing outcomes. Given the proposed role of mindfulness in emotion regulation (Guendelman et al., 2017), reduced mindfulness could be a major factor explaining the relationship between WLC and workers’ wellbeing. In line with theories and research on workers’ coping with affective events in the workplace (see Scheibe and Zacher, 2013), less efficient emotion regulation likely makes workers more vulnerable to the negative effects of WLC.

### Objectives and Hypotheses

In North America, the majority of research exploring WLC has studied the conflict between the demands of the workplace and family among English-speaking workers (e.g., Minnotte, 2012; Shen et al., 2015), but relatively little is known about the experiences of other cultural groups in North America. Using data collected from francophone participants, the largest linguistic group in Québec, Canada, the present study expands our understanding of the relationship between WLC and wellbeing with a population that is culturally distinct from most other provinces in Canada and jurisdictions in North America.

The aim of the present study is to examine the effects of WLC on positive and negative indicators of wellbeing by focusing on the interference of work with personal projects in a sample of francophone workers in Quebec, Canada. Two research questions act as a foundation for the present study: (1) how does WLC (i.e., time- and strain-based conflict between work and personal projects) relate to the positive mental health and workplace wellbeing of workers in Québec, Canada? (2) Is workplace mindfulness a mediator in the association between WLC and workers’ positive mental health and workplace wellbeing? It is further hypothesized that: (a) there are negative associations between time- and strain-based WLC and positive mental health and wellbeing at work; and (b) that these negative associations are explained by the fact that WLC is associated with reduced mindfulness, which in turn is related to lower levels of positive mental health and wellbeing at work.

## Materials and Methods

### Participants

A total of 330 participants based in Québec were surveyed for the purposes of the study, which is nested in a larger study focused on mental health in the workplace (see Meunier et al., 2018, for another published article from that study, focused on strengths use at work and work functioning). The collected sample had an average age of 33.6 years (*SD* = 11.64), with the lowest and maximum age being 18 and 70. A high proportion of the participants were women (82.2%), heterosexual (86.7%), born in Canada (86.1%), living with at least one more person in their household (78.3%), and had no children (71.5%). Participants were more likely to hold a university degree (56.3%) than a lower degree (34.9% of the participants held other forms of degrees beyond regular secondary diploma, such as college/trade school degrees). The majority of participants perceived themselves as comfortable financially or having sufficient income (73.7%), while 26.3% reported being poor or very poor. The majority of participants held office jobs (25.2%), while others held other positions as professionals (e.g., engineers, psychologists; 21.7%), blue-collar workers (9.9%), technicians (9.3%), managers/leaders (7.1%), or reported being in other jobs (26.7%). Of the participants, 51.4% worked full-time and 48.6% worked part-time.

### Procedure

The study was approved by two university Research Ethics Boards where the authors were employed at the time of the study (Université du Québec à Montréal REB #1044_2019; Wilfrid Laurier University REB #5063). De-identified data are available from the authors upon request. Participants were recruited through the help of community organizations within Québec, Canada, who posted an advertisement on their websites. The advertisement was also posted in other local classified online platforms (e.g., Kijiji, Craigslist) and on social media websites (e.g., Facebook). Interested participants had to complete screening questions to determine whether or not they met the inclusion criteria. If they did, participants could continue with completing the survey. Participants had to meet the following inclusion criteria: 18 years of age or older, working 10 hours or more, and be able to read and understand French (language of the majority in Québec). If participants met the inclusion criteria, the website redirected participants to the full online survey on a survey platform. All participants read a consent form and checked a box to indicate their consent to participate. At the end, participants were entered in a random draw to win one of six $50 Amazon gift cards. Given that the advertisement of the study was disseminated to numerous organizations who were invited to share the advertisements as widely as possible, the number of people that could have been reached or were actually reached by the advertisements are not known.

### Measures

All the following measures were provided to participants in French.

#### Positive Mental Health

Positive mental health was measured using the validated short form of the Mental Health Continuum (Keyes, 2002). The 14-item short-form scale, constructed by Keyes (2009), measures the frequency of experienced components of wellbeing within the previous month, including symptoms of emotional (e.g., happy), psychological (e.g., feeling that one likes most parts of their personality), and social (e.g., feeling of belonging to a community) wellbeing. Items are answered on a five-point Likert scale ranging from 0 (*never*) to 5 (*every day*). The Canadian Community Health Survey conducted by Statistics Canada (see Gilmour, 2014) uses the French version of the Mental Health Continuum Short Form, which is what we used in the presented study. In our study, the scale achieved a high level of internal consistency (α = 0.93).

#### Psychological Wellbeing at the Workplace

As a way of measuring psychological wellbeing within the workplace, a subscale was used (Thriving at Work) from a questionnaire originally developed in French by Dagenais-Desmarais and Savoie (2012). That subscale utilizes five items (e.g., “I find my job exciting,” “I find meaning in my work”) that participants answer on a six-point scale ranging from 0 (*disagree*) to 5 (*fully agree*). The scale had a high level of internal consistency (α = 0.86).

#### Work-Life Conflict

The perceived conflict of work commitments with workers’ personal projects was measured using an adapted six-item scale from Carlson et al. (2000) that was developed to measure work interference with family. The scale includes two subscales that measure the effect of strain- and time-based conflict in the workplace on family life. The original scale also includes a subscale related to an additional dimension (i.e., behavior-based conflicts), but it was not included for the purpose of the study. The scale was recently identified as one of the most commonly used measures to assess work-family conflict (Bansal and Agarwal, 2020). Carlson et al. (2000) have shown the reliability of each subscale, as well as their construct validity (e.g., time-based WLC related positively to the level of work involvement, strain-based WLC related negatively to family and life satisfaction).

In the current study, to capture work-personal project conflict (rather than general work-family conflict), items had to be altered to ask about the effect of work on participants’ own personal projects rather than on family life in general. Participants were thus asked to identify and focus on the most important personal projects in their life when answering the work-personal projects conflict scale. To accomplish this, an adaptation (i.e., a shortened version) of Little (1983)’s personal projects analysis approach was included, which asked participants to write down three personal projects, defined as daily life activities and goals (outside of work), that are important to the participants. It is important to note that the personal project measurement approach is intended to be modular and flexible, and the approach can – and should be – adapted to different research questions and contexts (Little and Balsari-Palsule, 2020), like in the current study.

The elicitation of personal projects followed an adapted French version (Houlfort and Sauvé, 2010) of the work-family interference strain- and time-based subscales developed by Carlson et al. (2000). Each subscale included three items that we adapted to focus on work-personal project conflict (rather than the focus on family in the original scale), for example “I am often so emotionally drained when I get home from work that it prevents me from doing my personal projects” (strain-based) and “I have to miss activities related to my personal projects due to the amount of time I must spend on work responsibilities” (time-based). Items were measured on a five-point Likert scale from 1 (*totally in disagreement*) to 5 (*totally in agreement*). Although the personal project-focused version of the strain- and time-based WLC subscales is a new measurement development proposed in the context of our study, our data supports the reliability and validity of this modified version of Carlson et al.’s (2000) subscales. We found a high level of consistency for both strain- and time-based conflict subscales (α = 0.84 and 0.88, respectively). Further, the correlations (see Table 1) between the subscales and outcome variables measured in the study follow an expected pattern (i.e., negative moderate correlations with positive mental health and thriving at work), which supports the concurrent validity of the subscales.

**TABLE 1:** Descriptive statistics and correlations between core variables and control variables (*n* = 288–330).

**Measures**	***N***	***M***	***SD***	**Skewness**	**Kurtosis**	1	2	3	4	5	6	7	8	9	10	11	12

**Core Variables**																	
1. Psychological wellbeing at work	330	3.74	0.91	–0.88	0.61	−											
2. Positive mental health	328	3.18	0.99	–0.45	–0.65	0.53**	−										
3. Mindfulness	321	4.37	0.99	–0.52	0.10	0.25**	0.41**	−									
4. Time-based conflict	290	3.09	1.18	–0.16	–0.96	−0.23**	−0.14*	−0.20**	−								
5. Strain-based conflict	291	3.22	1.14	–0.08	–0.81	−0.32**	−0.40**	−0.44**	0.50**	−							

**Control Variables**																	

6. Gender (1 = women; 2 = men)	325					–0.03	–0.05	–0.00	–0.02	–0.09	−						
7. Age	329	33.61	11.64	0.85	–0.26	0.08	0.04	0.08	–0.07	0.07	0.19**	−					
8. Perceived financial situation	320	2.15	0.66	0.28	0.24	–0.09	−0.22**	–0.09	0.05	0.11	0.06	–0.09	−				
9. Employment status (1 = full time; 2 = part-time)	321					–0.08	–0.05	0.02	0.02	–0.08	−0.12*	−0.44**	0.18**	−			
10. Duration (years) at current employer	322	5.37	6.74	2.19	5.66	0.12*	0.06	0.04	0.02	0.10	0.02	0.56**	−0.21**	−0.35**	−		
11. Neuroticism	330	4.06	1.50	0.02	–0.78	0.31**	0.43**	0.39**	–0.06	−0.29**	0.22**	0.16*	–0.10	–0.11	–0.01	−	
12. Perceived health status	330	6.36	1.99	–0.55	0.02	0.24**	0.43**	0.26**	−0.13*	−0.39**	0.02	0.05	−0.21**	–0.02	0.05	0.31**	−

*p ≤ 0.05; **p ≤ 0.01.

#### Mindfulness

Mindfulness in the workplace was measured using an adaptation of the Mindful Attention Awareness Scale, measuring dispositional mindfulness, developed by Brown and Ryan (2003). A translated French version was developed by Jermann et al. (2009). That scale, however, only assesses mindfulness in one’s life, generally speaking. The original English items had been altered by Dane and Brummel (2014) to refer specifically to mindfulness in the workplace, e.g., “When working… I find it difficult to stay focused on what’s happening in the present.” We applied the same adaptations made by Dane and Brummel (2014) to the French version (Jermann et al., 2009) of the original scale (Brown and Ryan, 2003) in order to obtain a French version focused on mindfulness in the workplace. The resulting score represents mindfulness at work, which, arguably, based on Dane and Brummel’s (2014) conceptualization, is dependent upon one’s level of dispositional mindfulness in life and other contextual factors affecting mindfulness specifically in one’s workplace. Participants answered the items using a six-point Likert scale ranging from 1 (*almost never*) to 6 (*almost always*). When implemented within our study, the workplace-adapted Mindfulness Attention Awareness Scale had a Cronbach’s alpha of 0.81.

#### Control Variables

In order to control for other variables that may influence the wellbeing variables, questions were added to account for different contextual and work-related factors. Gender was included as a binary variable, coding 1 for women and 2 for men. Participants also reported their age and answered a question asking to rate their perceived financial situation on a scale from 1 (*financially comfortable*) to 4 (*very poor*). Additionally, participants indicated if they work full-time (1) or part-time (2) and for how many years they have had the same position with the same employer (if they were not doing freelance work). Additionally, participants completed the Ten Item Personality Measure (TIPI; Gosling et al., 2003), which includes two items measuring each of the five personality traits of the Big 5 (extroversion, agreeableness, openness, conscientiousness, neuroticism), for a total of 10 items. As will be explained below, the neuroticism score (*r* = 0.40 between the two items of that subscale, which suggests adequate internal consistency, Briggs and Cheek, 1986) was used in the analysis. Finally, three items (α = 0.95) related to physical health from the PERMA-Profiler instrument (Butler and Kern, 2016) were included to obtain an indicator of the perceived health status of participants.

### Data Analysis

The hypotheses were explored by testing two full structural equation models using the Mplus software (Muthén and Muthén, 1998–2011). Each model focused on a different independent variable: one model with strain-based WLC and another one with time-based WLC. In each model, the measurement part included the observed indicators of WLC (i.e., conflict items) loading on the conflict latent construct, the mindfulness items loading on the mindfulness latent construct, and the psychological wellbeing at work and positive mental health items, each loading on their respective latent construct. The structural component of the model included unidirectional pathways from: (1) the WLC latent construct to the mindfulness latent construct, (2) the mindfulness latent construct to the psychological wellbeing at work latent construct and to the positive mental health latent construct, and (3) from the WLC construct to the psychological wellbeing at work latent construct and to the positive mental health latent construct (i.e., direct effects of the independent variables on the outcomes). In addition, to control for the effects of relevant control variables (age, gender, financial situation, full-time vs. part-time work, and length of current employment), pathways from each of these variables to the psychological wellbeing at work latent construct and to the positive mental health latent construct were included. The model also included a correlation between the two dependent latent constructs (psychological wellbeing at work and positive mental health). The software automatically allows the correlations between all independent variables (including control variables too) to be estimated.

The models were tested using Maximum Likelihood estimation with robust standard errors. Given that conflict variables were missing more than 5% of data (approximately 12% were missing on each), the Full Information Maximum Likelihood approach was used to deal with missing values. The Full Information Maximum Likelihood approach is recognized to be one of the best available means of reducing biases that could arise from missing values (Enders, 2010). The information available from all cases is used in the analysis without imputing data. To reduce missing data-related biases as much as possible, it is recommended to add auxiliary variables in the model in order to increase the likelihood of satisfying the missing condition at random (Enders, 2010). Auxiliary variables do not need to be of substantive interest, however, they need to be correlated with the variables with missing values or with the missingness of these variables. Enders (2010) suggests selecting auxiliary variables that have moderately large (or more) correlation coefficients. In our case, correlations were the largest (0.16 and more) for the TIPI neuroticism score and the perceived health status variable. As such, these variables were incorporated in the models using the Mplus dedicated auxiliary (*m*) command.

When testing the models, several indicators were considered to assess the adequacy of each model: Comparative Fit Index (CFI) and Tucker Lewis Index (TLI) ≥ 0.90; Root Mean Square Error of Approximation (RMSEA) ≤ 0.07; Standardized Root Mean Square Residual (SRMR) ≤ 0.08 (Hooper et al., 2008). For the initial testing of the first model (i.e., the model focused on time-based strain), modification indices were requested from the software. Modification indices help identify potential improvements that could be made to the model to provide better fit. Modification indices need to be considered with caution and theoretical considerations should be taken into account to make sure the final model’s substantive meaning is theoretically sound (Schumacker and Lomax, 2010).

Once the final model was obtained, the mediation hypothesis was tested. While it is recommended to implement bootstrapping of direct and indirect effects to assess mediation effects in a structural equation modeling environment (Cheung and Lau, 2008), it was not possible to perform bootstrapping in the current study given the inclusion of auxiliary variables: the (*m*) command necessary for the inclusion of such variables cannot be used in conjunction with bootstrapping in the Mplus software. As an alternative, the Monte Carlo Method for Assessing Mediation (MCMAM) was used (MacKinnon et al., 2004). It provides a confidence interval based on a Monte Carlo simulation from which to assess the significance of the indirect effect. The confidence interval was calculated using an online calculator developed by Selig and Preacher (2008), requesting 20,000 repetitions for the simulation.

## Results

### Descriptive Statistics and Univariate Correlations

As shown in Table 1, the main study variables were normally distributed, based on indices of skewness and kurtosis. The percentage of missing values for each variable varied between 0 and 12.12%. The two variables with the most missing values were the strain-based and time-based WLC variables. The personal project measure (Little, 1983) presented before these conflict items required participants to answer questions by entering a few words (i.e., short open-ended answers) describing their projects. It was observed that participants seemed more likely to skip these questions compared to other sections of the questionnaire, and as a result they could not answer the WLC questions because they referred to people’s personal projects.

Univariate correlations between the main study constructs were all significant at p ≤ 0.05 and are shown in Table 1. Mindfulness was positively correlated with both psychological wellbeing at work and positive mental health. Furthermore, positive mental health was positively correlated with psychological wellbeing at work. Time-based WLC was found to be negatively correlated with psychological wellbeing at work, positive mental health, and mindfulness. Strain-based WLC was positively correlated with time-based WLC, and negatively correlated with psychological wellbeing at work, positive mental health, and mindfulness.

The possibility of a common methods bias was examined by implementing the Harman’s single factor test using a) an exploratory approach (i.e., principal component analysis) and b) a confirmatory factor analysis approach. To do so, we conducted a principal component analysis in the SPSS software in which the individual items of relevant measures (model 1: mindfulness, time-based work-life conflict, positive mental health, thriving at work; model 2: same variables except that strain-based work-life conflict items were used instead of time-based items) were forced to load on one unrotated factor. The percentage of variance explained by the factor was, respectively, 33.4% and 33.9% for models 1 and 2. The fact that this percentage is below 50% suggests that common methods bias is not an issue (see Archimi et al., 2018). The same two models (i.e., models in which all items are loaded on a single shared construct) were also tested using the Mplus software and the fit indices were retrieved. The fit indices were not satisfying for model 1 and for model 2, respectively: χ2 (377) = 2529.66, TLI = 0.49; CFI = 0.53; RMSEA = 0.13 (90% CI [0.13, 0.14]); SRMR = 0.12, and χ2 (377) = 2407.55, TLI = 0.52; CFI = 0.56; RMSEA = 0.13 (90% CI [0.12, 0.13]); SRMR = 0.12. The lack of fit suggests that common methods bias is not an issue in this dataset (see Malhotra et al., 2006).

### Main Analysis

**FIGURE 1:**
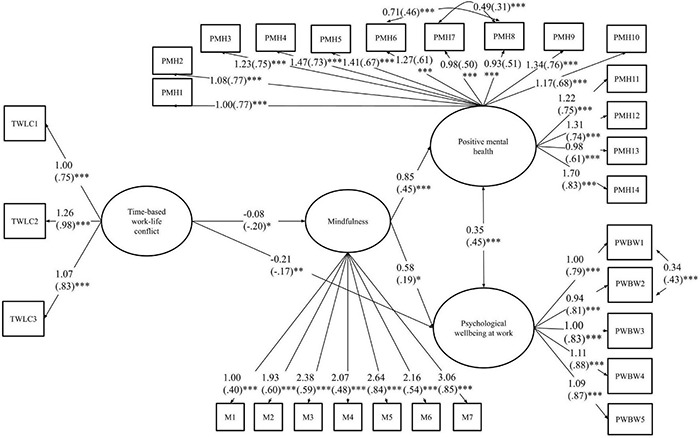
The (in)direct effect of time-based WLC on positive mental health and psychological wellbeing at work. The figure includes the unstandardized estimates accompanied by standardized estimates in brackets. Only significant links between core study variables are shown in the figure. Control variables described in the methods section were also included, but not represented to facilitate ease of reading. ^∗^*p* < 0.05, ^∗∗^*p* < 0.01, ^∗∗∗^*p* < 0.001.

The first tested model was the one including the time-based conflict construct as the independent variable. The initial fit of the model was relatively high, but not excellent: χ^2^ (501) = 1086.34, TLI = 0.87; CFI = 0.89; RMSEA = 0.06 (90% CI [0.06, 0.06]); SRMR = 0.06. Modification indices suggested adding correlation links between the sixth and the eighth item of the positive mental health measure, as well as between the seventh and the eighth item of that same measure. In addition, modification indices suggested adding a link between the first and the second item of the psychological wellbeing at work measure. The three identified items from the positive mental health measure were all related to the person’s relationship to society. As such, it made theoretical sense that these items would share additional variance beyond the general construct of wellbeing. The two identified items from psychological wellbeing at work both measured emotional responses in relation to work, i.e., excitement and satisfaction, thus it was understandable that these items would share additional variance beyond the general psychological wellbeing at work construct. These five suggested additional pathways were thus added. This resulted in better indicators of fit for the final model: χ^2^ (498) = 925.40, TLI = 0.92; CFI = 0.91; RMSEA = 0.05 (90% CI [0.05, 0.06]); SRMR = 0.06. That final model is shown in Figure 1. Only the significant pathways (*p* ≤ 0.05) between the core study variables are presented. As shown in the figure, all the observed indicators loaded as expected on their respective latent construct. There was a significant negative association between time-based WLC and mindfulness. That latent construct was associated positively with positive mental health and psychological wellbeing at work, which were positively associated with each other. While the direct effect of time-based WLC on positive mental health was not significant, there was a remaining significant direct, negative effect of time-based WLC on psychological wellbeing at work.

**FIGURE 2:**
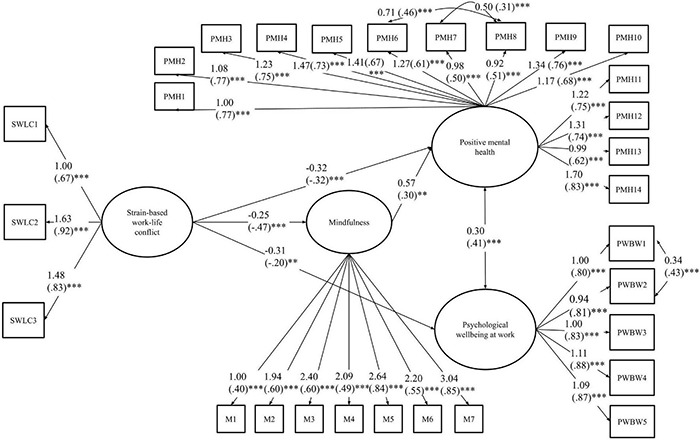
The (in)direct effect of strain-based WLC on positive mental health and psychological wellbeing at work. The figure includes the unstandardized estimates accompanied by standardized estimates in brackets. Only significant links between core study variables are shown in the figure. Control variables described in the methods section were also included, but not represented to facilitate ease of reading. ^∗^ *p* < 0.05, ^∗∗^*p* < 0.01, ^∗∗∗^*p* < 0.001.

For the second model, involving strain-based WLC as the independent latent construct, we integrated in the initial tested model the correlations that were added above based on the modification indices concerning positive mental health and psychological wellbeing at work items. The model showed satisfactory fit: χ^2^ (498) = 963.44, TLI = 0.90; CFI = 0.91; RMSEA = 0.05 (90% CI [0.05, 0.06]); SRMR = 0.06. No modifications were made. That final model is represented in Figure 2, with only significant pathways shown in the figure. As in the previous model, each indicator loaded as expected on their respective latent construct. There was a significant negative association between strain-based WLC and mindfulness. That latent construct was associated positively with positive mental health, but not associated with psychological wellbeing at work. There were significant direct negative effects of strain-based WLC on both positive mental health and psychological wellbeing at work.

For both models, MCMAM was used to assess the confidence intervals around the indirect effects of the mediator (i.e., mindfulness). For the indirect effect of time-based conflict on positive mental health through mindfulness, the Mplus-provided (unstandardized) estimate was −0.069 and the MCMAM-based confidence interval (95%) was [−0.118, −0.014]. Given that this interval did not include 0, we concluded that there was a significant negative effect of time-based conflict on positive mental health through mindfulness. Time-based conflict was related to lower mindfulness at work, and in turn, lower mindfulness was related to lower positive mental health. The fact that in the structural equation model (Figure 1) the remaining direct effect of time-based conflict on positive mental health was not significant suggests a complete mediation effect through mindfulness. The indirect effect of time-based conflict on psychological wellbeing at work was also significant and negative, but small: the Mplus-provided estimate was −0.047 and the MCMAM-based confidence interval was [−0.092, −0.002]. Time-based conflict was related to lower mindfulness at work, and in turn, lower mindfulness was related to lower psychological wellbeing at work. In that case, there was a remaining significant effect of time-based conflict on psychological wellbeing at work, suggesting a partial mediation effect.

For strain-based conflict, the indirect effect on positive mental health was significant and negative (Mplus-provided estimate: −0.141; MCMAM-based confidence interval: [−0.235, −0.047]). Strain-based conflict was related to lower mindfulness at work, and in turn, lower mindfulness was related to lower positive mental health. The fact that the remaining direct effect of time-based conflict on positive mental health remained significant (Figure 2) suggests a partial mediation effect. The indirect effect on psychological wellbeing at work was not significant (Mplus-provided estimate: −0.097; MCMAM-based confidence interval: [−0.213, 0.028]), which is consistent with the structural equation modeling results showing a non-significant pathway between mindfulness and psychological wellbeing at work in that model.

## Discussion

The study aimed to examine the associations of time- and strain-based work-life projects conflict with positive mental health and psychological wellbeing at work and to explore the role of mindfulness in these associations. Data were collected as part of an online survey conducted with a sample of francophone workers from Québec, Canada, allowing us to explore the topic of work-life conflict with a linguistic minority population within the larger North American context.

The first dependent variable considered was positive mental health, which represents symptoms of positive wellbeing at the emotional, psychological, and social levels (Keyes, 2005; Westerhof and Keyes, 2010; Provencher and Keyes, 2011). Both time- and strain-based conflicts of work with personal projects were found to be associated (directly and/or indirectly) with lower positive mental health. This suggests that, in addition to the associations demonstrated extensively in previous research between WLC and negative indicators of wellbeing (i.e., distress, anxiety, burnout, depression, Gisler et al., 2018; Neto et al., 2018), WLC could be detrimental to positive wellbeing. Previous research shows that positive and negative indicators of wellbeing tend to be correlated, although they do not necessarily have the same antecedents (Keyes, 2005; Karademas, 2007). Although no indicator of negative wellbeing was used in the present study, it is interesting that positive mental health was found to be associated negatively with WLC; it is expected based on previous research (Gisler et al., 2018; Neto et al., 2018) that WLC would also be associated with negative indicators (e.g., anxiety, depression) in our sample. Keyes (2013) argues that a comprehensive perspective on mental health and wellbeing needs to include an intention to study both positive and negative indicators, as they interact and can reinforce each other through time. More neglected by researchers and practitioners compared to negative indicators, positive mental health has been associated with reduced risk of developing future mental health issues (Keyes, 2013). In the workplace, workers’ positive mental health could be protective against work stressors (see Page et al., 2014). In contrast, reduced positive mental health related to work-life conflict could actually put a worker at risk of developing mental health issues in the future. Although research on workers’ positive mental health is still in its infancy, the results found in this study confirm and extend recent findings (Page et al., 2014; Fan et al., 2019; Hori et al., 2019). Particularly, Hori et al. (2019) identified a negative association of occupational stress (i.e., mental workload) with positive mental health; our results seem to suggest that workload and occupational stress could affect positive mental health through work-life conflict mechanisms.

The second dependent variable that we considered was psychological wellbeing at work, which also focuses on positive experiences, but specifically in the workplace context. Time- and strain-based WLC were both (directly and/or indirectly) related to lower psychological wellbeing at work. As highlighted in a review authored by Gisler et al. (2018), most studies have focused on the relationship between WLC and general indicators of psychological health outcomes, although some research has examined domain-specific indicators, such as those related to the workplace. Studies have highlighted the negative effects of WLC on job satisfaction, which in turn would affect general life satisfaction (Gisler et al., 2018). To our knowledge, our study is one of the first studies (or even the first study) to assess the association between WLC and workplace-specific wellbeing indicators beyond job satisfaction. In addition to including items related to feelings of satisfaction and excitement, the measure that we used (Thriving at Work subscale; Dagenais-Desmarais and Savoie, 2012) included items related to meaning, pride, and accomplishment in the workplace, which tap into what is called “eudaimonic wellbeing”, referring to individuals actualizing their potential and finding purpose. This aspect of wellbeing has only recently started to be recognized by organizational and work psychology researchers/practitioners as a central dimension of workers’ health (Bartels et al., 2019; Lysova et al., 2019). Our results contribute to this developing body of literature by identifying WLC as an important factor that may contribute to reduced psychological wellbeing at work. Further, the correlation we found between psychological wellbeing at work and positive mental health suggests that WLC may have synergistic effects, with workplace wellbeing and positive mental health being associated with each other and potentially reinforcing the influence of WLC.

Two interesting observations can be made when comparing the findings from the two models. A first observation is that strain-based WLC seemed to be more related to other variables in the model (e.g., wellbeing) than time-based WLC. This is consistent with findings from a meta-analysis of a small number of available studies in which it was found that strain-based conflict is more strongly related to exhaustion than time-based conflict (Reichl et al., 2014). Based on the interpretation provided by the authors of that meta-analysis, strain-based conflict is thought to directly impact the person’s stress system, while time-based conflict’s impact(s) on the stress system would have to be mediated through its effects on other life stressors, thus leading to some dilution of its measured effect on wellbeing (Reichl et al., 2014). Second, another observation is that the association between mindfulness and psychological wellbeing at work was not significant in the model focused on strain-based WLC while it was significant in the model focused on time-based WLC. However, a closer observation of the standardized estimates suggests only a small difference (magnitude of the difference: 0.06) in the strength of that association between the two models. This may represent a methodological artifact, or it could be due to the fact that inclusion of a different form of WLC has led to changes in the amount of remaining variance to explain in the psychological wellbeing at work variable.

The mediating role of mindfulness in the relationship between time- and strain-based WLC and positive mental health and psychological wellbeing at work was also examined. While most previous research has examined mindfulness as an intervention to reduce WLC or its effects (Michel et al., 2014; Kiburz et al., 2017), our study is the first one to explore the association between WLC and mindfulness (specifically at work) as a cognitive processing style in the workplace, and how in turn, this association relates to wellbeing outcomes. Overall the results confirm that WLC is associated with reduced mindfulness, which is associated with more negative wellbeing outcomes, i.e., reduced positive mental health and psychological wellbeing at work, in line with a few recent studies suggesting that workplace stressors could lead to decreased mindfulness (Davis et al., 2017; Hanley, 2017; Hülsheger et al., 2018; Crosswell et al., 2019).

In terms of the time-based dimension, there was no significant direct effect remaining in relation to positive mental health, suggesting a full mediation effect of mindfulness for that particular outcome. However, a significant remaining direct effect was found between time-based WLC and psychological wellbeing at work, after mindfulness was accounted for. Thus, mindfulness only partially mediated the effect of WLC on psychological wellbeing at work. For strain-based WLC, a similar pattern underlining a partial mediation effect was found for positive mental health. The measure of mindfulness used in the present study (Dane and Brummel, 2014) focused on mindfulness one demonstrates in work-related activities; it is possible that also considering the level of mindfulness that workers demonstrate at home or in their personal life could have led to a complete mediation effect. One could also argue that WLC is likely associated with workers’ wellbeing outcomes through multiple other pathways beyond reduced mindfulness. For instance, Bowen and Zhang (2020) suggest that work-life conflict may be associated with increased substance use and reduced sleep, which for example, we argue, could lead to lower wellbeing as well.

Importantly, WLC was framed around the notion of personal projects as workers were asked to reflect on the extent to which their work interferes with pursuing the projects that matter for them. As personal projects are thought to be highly idiosyncratic and central to people’s sense of who they are (Little, 1983, 1987), it is plausible that interference with personal projects has profound psychological effects on workers by hindering their potential to live in accordance with their personally valued ideals and goals and their authentic self. Future research should explore how the indirect effect of WLC through mindfulness interacts with other potential action mechanisms underlying the association between WLC and wellbeing outcomes, including goal-, value-, and identity-related processes.

### Limitations

The study has several limitations. Firstly, the study used a convenience sampling approach, limiting the generalizability of the results to the population of workers in Québec, Canada. In particular, the sample is relatively homogeneous in nature. For example, the sample consisted of young professionals (*M* = 33.6) who mostly were heterosexual and Canada-born. Thus, our results do not allow us to understand how time- and strain-based WLC and mindfulness may affect the wellbeing of workers that are not members of these dominant categories. For example, LGBTQ+ and newcomers may have different experiences and these are not necessarily represented in the current research (Ali et al., 2017; Kelliher et al., 2019). A recent Canadian study suggests a lack of knowledge of Indigenous perspectives on work-family conflict (Julien et al., 2017). More research with marginalized groups is needed. Additionally, different occupations and positions may present different stressors and expectations, altering WLC experiences and wellbeing (e.g., Higgins et al., 2008). This may not be appropriately captured within the presented study as only 9.9%, 9.3%, and 7.1% of participants reported being blue-collar workers, technicians, or managers/leaders, respectively.

Furthermore, the cross-sectional design of the study prevented us from asserting with certainty the directionality of the identified relationships (e.g., causality). Although the hypothesized directionality of the effects is plausible and based on our review of previous empirical and theoretical work, the study’s cross-sectional, correlational design explored the associations of time- and strain-based WLC with simultaneously measured mindfulness and wellbeing. Due to this, temporal precedence cannot be determined, nor can causality. It could be argued that mindfulness influences the perceived level of WLC (in addition to, or rather than, the opposite direction we put forward in this article). Future studies should investigate these relationships on a longitudinal basis by prospectively following workers over several months or years, with at least three measurement times to allow for a fully longitudinal cross-lagged mediation model to be tested (Selig and Preacher, 2009).

As it may be argued that some factors (e.g., change in leadership, working for a new company, completing a personal project) may lead to concomitant variations in both WLC and mindfulness, future research should also explore potential third variables that could account for the relationship between WLC and mindfulness in order to better establish the causal pathway between these two constructs. Additionally, the current sample size prevented us from examining how both time- and strain-based WLC related to outcomes within a single model. Important knowledge may be gained by incorporating both in a single analysis in order to capture and better understand their potentially interactive impacts on workers’ wellbeing. In the current study, the ratio of the sample size on the number of free parameters in each tested model was approximately three, which is lower than the recommended minimum guidelines (i.e., ratio of 5 or 10, Wang and Wang, 2019). This could have reduced statistical power, and future research is important to further establish the stability of the results.

More research is also needed to better understand how pursuing personal projects may actually play a moderating role on the relationship between WLC and wellbeing. From an existential perspective, it could be argued that if one’s personal projects are meaningful, this could buffer the effect of work-related stressors on the person’s health and wellbeing, given that meaning in life has been identified to be a buffering factor against the impact of stress (Halama, 2014).

The interference of personal projects on work should also be considered. Little’s (1983) personal projects analysis includes a cross-impact matrix that could be adapted in the future to elicit workers’ personal and occupational projects and the reciprocal influence/interference between these goals based on participants’ perceptions (see Wiese and Salmela-Aro, 2008 for an example focused on work and family goals). While the use of a personal project analysis-inspired approach proved to be worthwhile in the present study, the open-ended component (i.e., project list elicitation) may have been perceived to be a burden to some participants, explaining the higher level of missing data on the project-related WLC questions. Future research should explore innovative ways to promote greater participation on these projects-related questions in online survey contexts.

### Practical Implications

The study provides cross-sectional evidence that WLC is associated with workers’ lower positive wellbeing in their general life, as well as in the workplace. Furthermore, the results suggest that reduced mindfulness may play a central role in the relationship between WLC and wellbeing outcomes. While more research is needed to firmly establish the directionality of the effects observed in the study, the findings allow us to formulate preliminary recommendations for policymakers, occupational health professionals, and leaders. First, the study adds to the demonstration of the importance for policymakers and workplace leaders to implement programs and policies to institutionalize practices that could help reduce WLC (e.g., allowing flexwork, reducing workload; Higgins et al., 2008); not only can WLC be associated with risks of mental health issues as demonstrated in previous research, but it can also affect workers’ capacity to live a fulfilling personal and professional life. Second, mindfulness-based interventions (e.g., Wolever et al., 2012; Grégoire and Lachance, 2015; Huang et al., 2015) offered in the workplace may be beneficial for workers as they could counteract the negative effects of WLC on workers’ wellbeing by promoting emotion regulation skills (Guendelman et al., 2017). Third, our results suggest that employers should consider devoting more attention and time to listening to workers’ concerns about the effects of their work on the pursuit of the projects that matter in their personal lives. By learning about workers’ personal goals and aspirations, organizations would be better positioned to co-design innovative and efficient means to reduce work-life conflict and to meaningfully support workers in developing themselves outside of work.

## Data Availability Statement

The datasets generated for this study are available on request to the corresponding author.

## Ethics Statement

The study was reviewed and approved by the Comité Institutionnel d’Éthique de la Recherche Avec des Êtres Humains from Université du Québec à Montréal and by Wilfrid Laurier University’s Research Ethics Board. The patients/participants provided their written informed consent to participate in this study.

## Author Contributions

TP wrote most parts of the manuscript, based on his original research idea. SC supervised manuscript writing, conducted the analysis with TP, and wrote the Results section. SM co-developed the study with SC, supervised data collection/cleaning, and helped to develop the knowledge mobilization plan. All authors contributed to the article and approved the submitted version.

## Conflict of Interest

The authors declare that the research was conducted in the absence of any commercial or financial relationships that could be construed as a potential conflict of interest.

## Publisher’s Note

All claims expressed in this article are solely those of the authors and do not necessarily represent those of their affiliated organizations, or those of the publisher, the editors and the reviewers. Any product that may be evaluated in this article, or claim that may be made by its manufacturer, is not guaranteed or endorsed by the publisher.
